# Supplementary material for: Glucose-Lowering Drugs and Primary Prevention of Chronic Kidney Disease in Type 2 Diabetes Patients: A Real-World Primary Care Study
Source: Pharmaceuticals (Basel). 2024 Sep 29;17(10):1299. doi: 10.3390/ph17101299 (PMC11510410; doi:10.3390/ph17101299)
Supplement: Supplementary file 1 [file pharmaceuticals-17-01299-s001.zip › pharmaceuticals-3189904-supplementary.pdf]

GLUCOSE-LOWERING DRUGS AND PRIMARY PREVENTION OF CHRONIC KIDNEY DISEASE IN TYPE 2 DIABETES PATIENTS: A REAL-WORLD PRIMARY CARE STUDY

Antonio Rodríguez-Miguel, Beatriz Fernández-Fernández, Alberto Ortiz, Miguel Gil, Sara Rodríguez-Martín, Gema Ruiz-Hurtado, Encarnación Fernández-Antón, Luis Miguel Ruilope, and Francisco J. de Abajo

SUPPLEMENTARY MATERIALS

Supplementary Methods

All potential confounding factors were selected by expert criteria, thus avoiding the use of data driven methods. Potential confounding factors included the presence of comorbidities at index date as well as the use of comedications from index date backwards. This way, to obtain the fully-adjusted odds ratios, we included in the conditional logistic model the following comorbidities: age at index date, sex, years of follow-up since entry in the type 2 diabetes cohort (T2DM-cohort), alcohol abuse (recorded as such by the physician), obesity (recorded as such by the physician or a by a record of BMI ≥30 kg/m2), use of tobacco, prior isolated pathological records of eGFR, prior isolated pathological records of proteinuria or albuminuria, hypertension, dyslipidemia, atrial fibrillation, ischemic heart diseases (including acute myocardial infarction and angina pectoris), heart failure, gout (recorded as such by the physician or by a prescription of urate lowering drugs), hiperuricemia (non-gout), stroke (including ischemic, hemorraghic or lacunar stroke, and transient ischemic attack), peripheral artery disease, hyperparathyroidism, osteoporosis, hematuria (recorded as such by the phsysician), and hyperkalemia (recorded as such by the physician), as well as the

following comedications: antihypertensives (including alpha-adrenoreceptor antagonists, beta blocking agents, calcium channel blockers, angiotensin converting enzyme inhibitors, angiotensin II receptor blockers, and renin-inhibitors), diuretics (including high-ceiling, low-ceiling thiazides, low-ceiling excluding thiazides, direct potassium-sparing agents and, mineralocorticoid receptor antagonists), antiplatelet drugs (including COX-1 inhibitors, $P2Y_{12}$ receptor blockers, others), oral anticoagulants (including vitamin K antagonists, direct thrombin inhibitors, direct factor Xa inhibitors), heparins, class I and III antiarrhythmics, nonsteroidal anti-inflammatory drugs, paracetamol, metamizole, symptomatic slow-action drugs for osteoarthritis, opioids, glucocorticoids for systemic use, proton-pump inhibitors, H2-receptor antagonists, immunosuppressants, benzodiazepines, antidepressants, antiepileptics, anti-Parkinson drugs, antipsychotics, vitamin D and calcium (alone or in combination), and colchicine.

Supplementary Table S1. Association of new-onset chronic kidney disease with the current use of different glucose-lowering drugs, and effect of duration of treatment.

	CASES (*n*=89 075)	CONTROLS (*n*=442 216)	Follow-up in controls, median (IQR), in years	Age and Sex ADJUSTED OR (95%CI)[a]	FULLY ADJUSTED OR (95%CI)[a,b]
**Biguanides:**					
Any duration	69 412 (77.9)	327 044 (74.0)	3.23 (1.41 – 5.84)	1.31 (1.28 – 1.35)	1.20 (1.17 – 1.23)
<3 years	28 524 (32.0)	144 631 (32.7)		1.22 (1.19 – 1.25)	1.18 (1.15 – 1.21)
≥3 years	40 888 (45.9)	182 413 (41.3)		1.42 (1.38 – 1.45)	1.22 (1.18 – 1.25)
**Sulfonylureas:**					
Any duration	20 388 (22.9)	99 405 (22.5)	3.13 (1.40 – 5.73)	1.06 (1.04 – 1.08)	1.08 (1.06- 1.10)
<3 years	11 005 (12.4)	52 466 (11.9)		1.08 (1.06 – 1.11)	1.10 (1.07 – 1.13)
≥3 years	9383 (10.5)	46 939 (10.6)		1.03 (1.00 – 1.05)	1.06 (1.03 – 1.09)
**Alpha glucosidase inhibitors:**					
Any duration	2020 (2.27)	10 873 (2.46)	2.17 (0.96 – 4.06)	0.92 (0.88 – 0.97)	1.00 (0.95 – 1.06)
<3 years	856 (0.96)	4784 (1.08)		0.89 (0.82 – 0.95)	0.94 (0.87 – 1.01)
≥3 years	1164 (1.31)	6089 (1.38)		0.95 (0.90 – 1.02)	1.06 (0.99 – 1.13)
**Thiazolidinediones:**					
Any duration	2156 (2.42)	8432 (1.91)	2.97 (1.43 – 4.98)	1.30 (1.24 – 1.36)	1.13 (1.08 – 1.19)
<3 years	1280 (1.44)	5195 (1.17)		1.25 (1.18 – 1.33)	1.08 (1.01 – 1.15)
≥3 years	876 (0.98)	3237 (0.73)		1.37 (1.27 – 1.48)	1.22 (1.12 – 1.31)
**DPP-4 inhibitors:**					
Any duration	26 724 (30.0)	112 830 (25.5)	4.49 (2.24 – 7.32)	1.29 (1.27 – 1.32)	1.16 (1.14 – 1.18)
<3 years	17 462 (19.6)	75 076 (17.0)		1.27 (1.24 – 1.29)	1.14 (1.12 – 1.17)
≥3 years	9262 (10.4)	37 754 (8.54)		1.36 (1.32 – 1.39)	1.20 (1.17 – 1.24)
**GLP-1 receptor agonists:**					
Any duration	1382 (1.55)	5131 (1.16)	4.83 (2.63 – 8.01)	1.35 (1.27 – 1.43)	0.94 (0.88 – 1.01)
<3 years	1159 (1.30)	4166 (0.94)		1.39 (1.30 – 1.48)	0.96 (0.90 – 1.03)
≥3 years	223 (0.25)	965 (0.22)		1.16 (1.00 – 1.34)	**0.85 (0.73 – 0.99)**
**SGLT-2 inhibitors:**					
Any duration	3025 (3.40)	12 345 (2.79)	5.50 (3.11 – 8.59)	1.24 (1.19 – 1.30)	1.07 (1.02 – 1.11)
<3 years	2887 (3.24)	11 629 (2.63)		1.26 (1.21 – 1.31)	1.07 (1.02 – 1.12)
≥3 years	138 (0.15)	716 (0.16)		0.99 (0.82 – 1.20)	**0.89 (0.74 – 1.08)**
**Glinides:**					
Any duration	9265 (10.4)	36 107 (8.17)	3.74 (1.72 – 6.51)	1.32 (1.29 – 1.36)	1.22 (1.19 – 1.25)
<3 years	4840 (5.43)	19 396 (4.39)		1.29 (1.24 – 1.33)	1.18 (1.14 – 1.22)
≥3 years	4425 (4.97)	16 711 (3.78)		1.37 (1.32 – 1.41)	1.26 (1.21 – 1.30)
**Insulin:**					
Any duration	16 266 (18.3)	66 155 (15.0)	4.54 (2.11 – 7.65)	1.27 (1.25 – 1.29)	1.14 (1.12 – 1.17)
<3 years	7438 (8.35)	29 762 (6.73)		1.29 (1.25 – 1.32)	1.14 (1.10 – 1.17)
≥3 years	8828 (5.31)	36 393 (8.23)		1.25 (1.22 – 1.28)	1.14 (1.11 – 1.17)

IQR: interquartile range; OR: odds ratio; CI: confidence interval; DPP-4: dipeptidyl peptidase-4; GLP-1: glucagon-like peptide-1; SGLT-2: sodium-glucose cotransporter 2

[a] The category of reference is the non-use of each subgroup; [b] Adjusted for all the variables in the Table 2 (excepting CKD stage) plus the following comedication in the year before index date: antihypertensives

(including alpha-adrenoreceptor antagonists, beta blocking agents, calcium channel blockers, angiotensin converting enzyme inhibitors, angiotensin II receptor blockers, and renin-inhibitors), diuretics (including high-ceiling, low-ceiling thiazides, low-ceiling excluding thiazides, direct potassium-sparing agents and, mineralocorticoid receptor antagonists), antiplatelet drugs (including COX-1 inhibitors, P2Y12 receptor blockers, others), oral anticoagulants (including vitamin K antagonists, direct thrombin inhibitors, direct factor Xa inhibitors), heparins, class I and III antiarrhythmics, nonsteroidal anti-inflammatory drugs, paracetamol, metamizole, symptomatic slow-action drugs for osteoarthritis, opioids, glucocorticoids for systemic use, proton-pump inhibitors, H2-receptor antagonists, immunosuppressants, benzodiazepines, antidepressants, antiepileptics, anti-Parkinson drugs, antipsychotics, vitamin D and calcium (alone or in combination), and colchicine.

Supplementary Table S2. Association of new-onset chronic kidney insufficiency (stages G3 to G5) with the current use of different glucose-lowering drugs, and effect of duration of treatment

	CASES (*n*=44 447)	CONTROLS (*n*=221 157)	Follow-up in controls, median (IQR), in years	Age and Sex ADJUSTED OR (95%CI)[a]	FULLY ADJUSTED OR (95%CI)[a,b]
**Biguanides:**					
Any duration	33 553 (75.5)	160 198 (72.4)	3.31 (1.45 – 5.96)	1.28 (1.23 – 1.32)	1.11 (1.07 – 1.15)
<3 years	12 765 (28.7)	68 046 (30.8)		1.14 (1.10 – 1.19)	1.08 (1.04 – 1.12)
≥3 years	20 788 (46.8)	92 152 (41.7)		1.41 (1.36 – 1.46)	1.14 (1.09 – 1.18)
**Sulfonylureas:**					
Any duration	10 978 (24.7)	55 520 (25.1)	3.05 (1.35 – 5.60)	1.01 (0.98 – 1.03)	1.01 (0.98 – 1.04)
<3 years	5849 (13.2)	28 657 (13.0)		1.04 (1.01 – 1.07)	1.04 (1.00 – 1.08)
≥3 years	5129 (11.5)	26 863 (12.2)		0.97 (0.94 – 1.00)	0.97 (0.93 – 1.00)
**Alpha glucosidase inhibitors:**					
Any duration	1126 (2.53)	6422 (2.90)	2.11 (0.96 – 3.97)	0.87 (0.81 – 0.93)	0.93 (0.87 – 1.00)
<3 years	466 (1.05)	2870 (1.30)		0.80 (0.72 – 0.88)	0.82 (0.74 – 0.92)
≥3 years	660 (1.48)	3552 (1.61)		0.92 (0.85 – 1.00)	1.03 (0.94 – 1.13)
**Thiazolidinediones:**					
Any duration	1124 (2.53)	4411 (1.99)	2.89 (1.34 – 4.88)	1.30 (1.21 – 1.39)	1.14 (1.06 – 1.23)
<3 years	664 (1.49)	2737 (1.24)		1.24 (1.13 – 1.35)	1.06 (0.96 – 1.17)
≥3 years	460 (1.03)	1674 (0.76)		1.40 (1.26 – 1.55)	1.26 (1.12 – 1.41)
**DPP-4 inhibitors:**					
Any duration	13 249 (29.8)	54 444 (24.6)	4.62 (2.30 – 7.41)	1.36 (1.32 – 1.39)	1.22 (1.19 – 1.26)
<3 years	8725 (19.6)	36 224 (16.4)		1.34 (1.30 – 1.37)	1.20 (1.17 – 1.24)
≥3 years	4524 (10.2)	18 220 (8.24)		1.40 (1.35 – 1.46)	1.25 (1.19 – 1.30)
**GLP-1 RA:**					
Any duration	517 (1.16)	1777 (0.80)	5.18 (2.78 – 8.53)	1.47 (1.33 – 1.62)	0.91 (0.81 – 1.02)
<3 years	434 (0.98)	1399 (0.63)		1.56 (1.40 – 1.74)	0.96 (0.85 – 1.08)
≥3 years	83 (0.19)	378 (0.17)		1.11 (0.87 – 1.41)	**0.72 (0.56 – 0.94)**
**SGLT-2 inhibitors:**					
Any duration	1179 (2.65)	4897 (2.21)	5.83 (3.37 – 9.07)	1.23 (1.15 – 1.31)	1.04 (0.97 – 1.12)
<3 years	1137 (2.56)	4595 (2.08)		1.26 (1.18 – 1.35)	1.06 (0.98 – 1.14)
≥3 years	42 (0.09)	302 (0.14)		0.71 (0.51 – 0.99)	**0.64 (0.46 – 0.91)**
**Glinides:**					
Any duration	4647 (10.5)	19 264 (8.71)	3.64 (1.68 – 6.36)	1.24 (1.20 – 1.28)	1.10 (1.06 – 1.14)
<3 years	2464 (5.54)	10 222 (4.62)		1.24 (1.18 – 1.29)	1.09 (1.03 – 1.15)
≥3 years	2183 (4.91)	9042 (4.09)		1.24 (1.18 – 1.30)	1.09 (1.03 – 1.15)
**Insulin:**					
Any duration	8377 (18.9)	34 903 (15.8)	4.47 (2.07 – 7.53)	1.24 (1.21 – 1.27)	1.04 (1.01 – 1.08)
<3 years	3747 (8.43)	15 372 (6.95)		1.25 (1.21 – 1.30)	1.06 (1.02 – 1.11)
≥3 years	4630 (10.4)	19 531 (8.83)		1.22 (1.18 – 1.27)	1.02 (0.98 – 1.07)

IQR: interquartile range; OR: odds ratio; CI: confidence interval; DPP-4: dipeptidyl peptidase-4; GLP-1: glucagon-like peptide-1; SGLT-2: sodium-glucose cotransporter 2

[a] The category of reference is the non-use of each subgroup; [b] Adjusted for all the variables in the Table 2 (excepting CKD stage) plus the following comedication in the year before index date: antihypertensives (including alpha-adrenoreceptor antagonists, beta blocking agents, calcium channel blockers, angiotensin

converting enzyme inhibitors, angiotensin II receptor blockers, and renin-inhibitors), diuretics (including high-ceiling, low-ceiling thiazides, low-ceiling excluding thiazides, direct potassium-sparing agents and, mineralocorticoid receptor antagonists), antiplatelet drugs (including COX-1 inhibitors, P2Y12 receptor blockers, others), oral anticoagulants (including vitamin K antagonists, direct thrombin inhibitors, direct factor Xa inhibitors), heparins, class I and III antiarrhythmics, nonsteroidal anti-inflammatory drugs, paracetamol, metamizole, symptomatic slow-action drugs for osteoarthritis, opioids, glucocorticoids for systemic use, proton-pump inhibitors, H2-receptor antagonists, immunosuppressants, benzodiazepines, antidepressants, antiepileptics, anti-Parkinson drugs, antipsychotics, vitamin D and calcium (alone or in combination), and colchicine.
